# Non-Persistence With Antiplatelet Medications Among Older Patients With Peripheral Arterial Disease

**DOI:** 10.3389/fphar.2021.687549

**Published:** 2021-05-19

**Authors:** Martin Wawruch, Jan Murin, Tomas Tesar, Martina Paduchova, Miriam Petrova, Denisa Celovska, Beata Havelkova, Michal Trnka, Emma Aarnio

**Affiliations:** ^1^ Institute of Pharmacology and Clinical Pharmacology, Faculty of Medicine, Comenius University, Bratislava, Slovakia; ^2^ 1st Department of Internal Medicine, Faculty of Medicine, Comenius University, Bratislava, Slovakia; ^3^ Department of Organization and Management of Pharmacy, Faculty of Pharmacy, Comenius University, Bratislava, Slovakia; ^4^ Department of Angiology, Health Centre, Trnava, Slovakia; ^5^ General Health Insurance Company, Bratislava, Slovakia; ^6^ Institute of Medical Physics, Biophysics, Informatics and Telemedicine, Faculty of Medicine, Comenius University, Bratislava, Slovakia; ^7^ Institute of Biomedicine, University of Turku, Turku, Finland; ^8^ School of Pharmacy, University of Eastern Finland, Kuopio, Finland

**Keywords:** peripheral arterial disease, antiplatelet medications, non-persistence, discontinuation, atrial fibrillation, anxiety disorders

## Abstract

**Introduction:** Antiplatelet therapy needs to be administered life-long in patients with peripheral arterial disease (PAD). Our study was aimed at 1) the analysis of non-persistence with antiplatelet medication in older PAD patients and 2) identification of patient- and medication-related characteristics associated with non-persistence.

**Methods:** The study data was retrieved from the database of the General Health Insurance Company. The study cohort of 9,178 patients aged ≥ 65 years and treated with antiplatelet medications was selected from 21,433 patients in whom PAD was newly diagnosed between 01/2012 and 12/2012. Patients with a 6 months treatment gap without antiplatelet medication prescription were classified as non-persistent. Characteristics associated with non-persistence were identified using the Cox regression.

**Results:** At the end of the 5 years follow-up, 3,032 (33.0%) patients were non-persistent. Age, history of ischemic stroke or myocardial infarction, clopidogrel or combination of aspirin with clopidogrel used at the index date, higher co-payment, general practitioner as index prescriber and higher overall number of medications were associated with persistence, whereas female sex, atrial fibrillation, anxiety disorders, bronchial asthma/chronic obstructive pulmonary disease, being a new antiplatelet medication user (therapy initiated in association with PAD diagnosis), and use of anticoagulants or antiarrhythmic agents were associated with non-persistence.

**Conclusion:** In patients with an increased probability of non-persistence, an increased attention should be paid to improvement of persistence.

## Introduction

Our manuscript is focused on older patients with peripheral arterial disease (PAD) of lower limbs, a chronic atherosclerotic disease affecting the peripheral vasculature of lower limbs. It is associated with limb-related symptoms and complications such as intermittent claudication, ischemic rest pain, and critical limb ischemia. PAD may result in gangrene of the affected limb requiring amputation. Since atherosclerosis represents a generalized process affecting the whole cardiovascular (CV) system, PAD is associated with an increased risk of CV events (ischemic stroke, myocardial infarction (MI) and CV death) (Agrawal and Eberhardt, 2015; Bonaca and Creager, 2015; Morley et al., 2018). PAD is a relatively frequent disease, its prevalence increasing with advancing age. According to the systematic review by Fowkes et al. (2013), 202 million people were affected with PAD globally in 2010, 69.7% of them in low- or middle-income countries. In high-income countries, the prevalence of PAD at age 45–49 years was 5.3% among women and 5.4% among men and, at age 85–89 years, it was 18.4% among women and 18.8% among men.

Besides the management of modifiable risk factors (smoking cessation, pharmacologic treatment of high blood pressure, increased blood glucose levels, and dyslipoproteinemia), PAD treatment includes administration of antiplatelet agents, inhibitors of angiotensin-converting enzyme**/**angiotensin receptor blockers and statins. Platelet hyperaggregability in PAD patients as well as an important role of platelets in the atherosclerotic process justify the use of antiplatelet agents in the treatment of PAD (Aboyans et al., 2018; Bevan and White Solaru, 2020).

Adherence to antiplatelet medication represents the basic requirement for successful treatment of PAD patients. Although medications used in treatment of CV diseases have improved significantly, adherence to these medications remains unsatisfactory (Xu et al., 2020). The issue of medication adherence is particularly relevant in case of asymptomatic conditions (e.g., treatment of CV risk factors) (Burnier, 2019). Adherence includes three interrelated phases: initiation, implementation and persistence. Initiation represents the start of using the prescribed medication. Implementation reflects the extent to which a patient´s actual dosing corresponds to the prescribed dosing (from initiation until the last dose). Persistence refers to the length of time between initiation and discontinuation (Vrijens et al., 2012; De Geest et al., 2018). Since antiplatelet therapy needs to be administered life-long in PAD patients, we focused our study on the issue of non-persistence with this medication.

In the literature there is a lack of information about adherence to medications used in secondary prevention in older PAD patients. The study by Qvist et al. (2019) evaluated adherence to antiplatelet and statin therapy; however, besides PAD patients, it included also subjects with abdominal aortic aneurysm, and the age of participants was limited to the range of 65–74 years. Persistence was defined as an absence of treatment gap > 100 days between two prescription renewals. No predictors of persistence were found in the study. To fill in this gap in the literature and describe patterns of persistence with antiplatelet therapy in older PAD patients, our study was aimed at 1) the analysis of non-persistence and 2) identification of patient- and medication-related characteristics associated with non-persistence. To the best of our knowledge, our study is the first one to evaluate these issues to such an extent.

## Methods

### Database and Study Population

**FIGURE 1:**
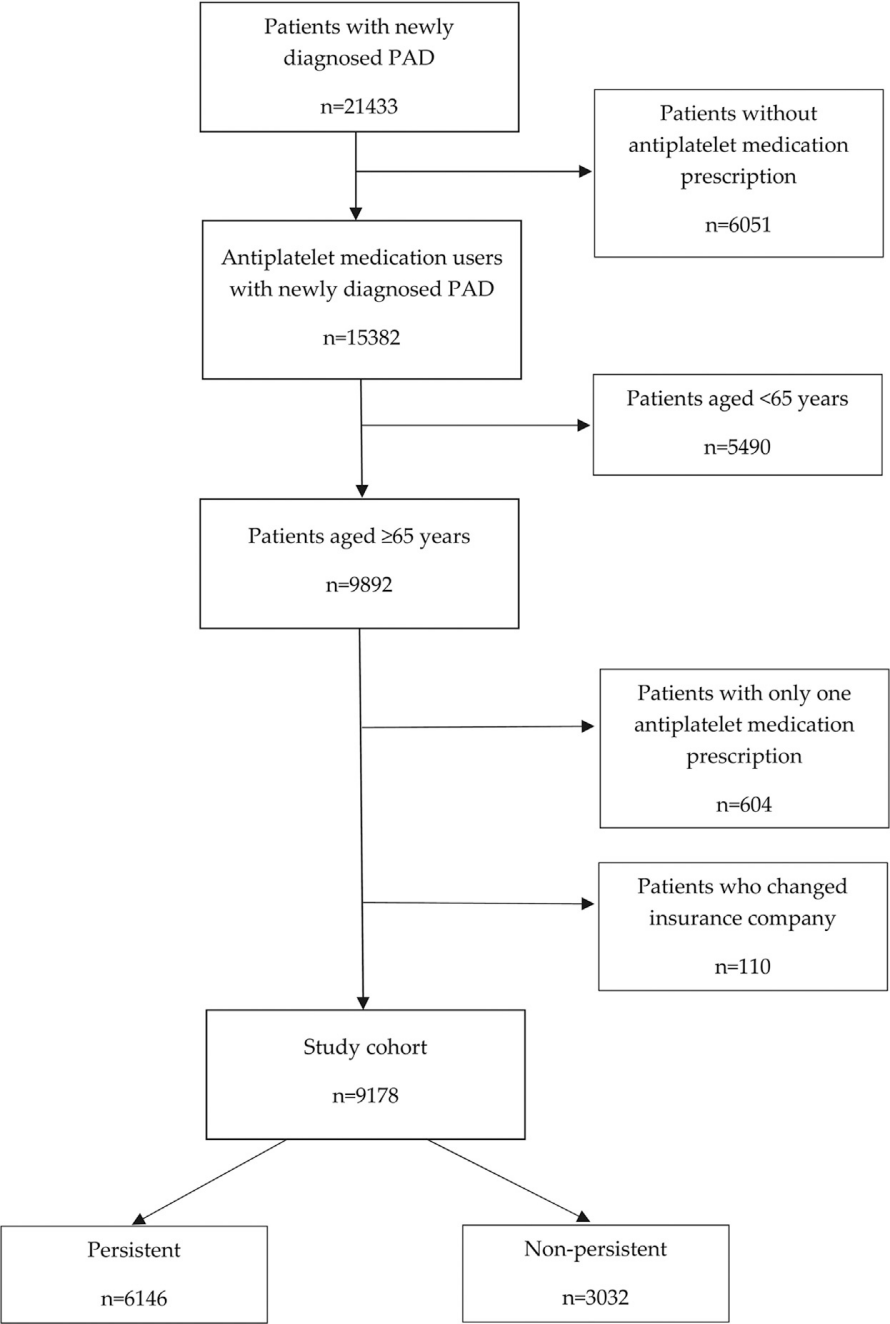
Flow chart of the study cohort (*n* = 9,178).

The study data was retrieved from the database of the General Health Insurance Company. It is the largest health insurance provider in Slovakia covering approximately 63% of the population. In this database, 21,433 patients in whom PAD was newly diagnosed between January 1 and December 31, 2012 were identified. Among the patients in this database, antiplatelet medication use was recorded in 15,382 patients. Out of these patients, those aged ≥ 65 years (*n* = 9,892) were selected. Patients with only one antiplatelet medication prescription during the 5 years follow-up period (*n* = 604) and those who changed their health insurance company (*n* = 110) were excluded. After the exclusion of these patients, there remained a sample of 9,178 patients used as the study cohort for further evaluations (Figure 1). This database of 21,433 patients represented a source of data in our previous study focused on non-persistence with statin treatment in older patients with PAD (Wawruch et al., 2019). In Slovakia, aspirin is available as an over-the-counter drug, but in case of diseases in whose treatment aspirin is fully indicated (e.g., PAD), it is prescribed by a physician. Consequently, its use in PAD patients can be traced via registers.

### Analysis of Non-Persistence

The index date of our retrospective cohort study was the date of the first dispensation of antiplatelet medication at a pharmacy after the diagnosis of PAD. From the index date, patients were followed for 5 years or up to the date of their death if it occurred during the follow-up period. Patients who died were censored to avoid their misclassification as non-persistent subjects.

Non-persistence was identified according to the treatment gap period which was defined as a 6 months period without any antiplatelet medication prescription observed after the estimated date of the last day covered by the last package of the prescribed medication. All tablets in previous packages were considered when calculating the length of the period covered by medication (i.e., tablets carried over in case of early prescriptions). The start of non-persistence was set at the first day after the end of the period covered by the prescribed medication, i.e., the first day of treatment gap. Antiplatelet medications were considered as a medication group, i.e., persistence with particular antiplatelet agents, besides the initial treatment, was not examined. Except for ticlopidine, dosing of one tablet per day was considered to calculate the number of tablets of antiplatelet medications needed for a certain time period. In case of ticlopidine, twice daily administration was considered. Patients with a treatment gap period were classified as non-persistent and those without such period were considered as persistent.

### Analysis of Factors Associated With Non-Persistence

Data on patient- and medication-related characteristics, evaluated as factors potentially associated with non-persistence, were collected at the time of inclusion in the study cohort. The following characteristics were analyzed:

a) Socio-demographic characteristics: age, gender, university education, and employment.
b) History of CV events: ischemic stroke, transient ischemic attack (TIA), and MI during 5 years before the index date.
c) Number of comorbid conditions and particular comorbidities. Data on comorbid conditions were collected in accordance with the 10th revision of the International Classification of Diseases (ICD-10, 1992) (Supplementary Table S1).
d) Antiplatelet medication-associated characteristics: initially (i.e., on the index date) administered antiplatelet agent(s), whether the patient was a new (antiplatelet treatment initiated in association with PAD diagnosis) or prevalent (administered already before PAD diagnosis) user of antiplatelet medication, patient’s co-payment per one month, and whether the antiplatelet medication was prescribed initially after the PAD diagnosis by a general practitioner or a specialist. To identify new users, a period of at least 2 years without antiplatelet medication prescription before PAD diagnosis was required.
e) The overall number of medications, the number of CV co-medications and particular CV medications identified according to ATC codes (Guidelines for ATC Classification and DDD Assignment, 2018) (Supplementary Table S2)

### Statistical Analysis

Continuous variables were expressed as means ± standard deviations, and categorical variables as frequencies and percentages.

Categorical variables were compared between persistent and non-persistent patients using the χ^2^-test. The Mann-Whitney U test was applied to compare continuous variables between the two patient groups. This non-parametric test was used because of non-Gaussian distribution of evaluated variables. The distribution of continuous variables was analyzed by the Kolmogorov-Smirnov test.

The Life table analysis was used to identify numbers and proportions of patients who became non-persistent during each particular year of the 5 years follow-up period. To analyze the development of non-persistence during the follow-up period in relation to the initially used antiplatelet medication, and to clearly illustrate the differences in non-persistence among patients with particular antiplatelet agents, the Kaplan-Meier model was applied. To identify the significance of these differences, the log-rank test was used. To identify patient- and medication-related characteristics associated with non-persistence, the Cox proportional hazards model was applied. Hazard ratios and corresponding 95% confidence intervals were determined for each characteristic. The Cox regression included all variables evaluated as factors potentially associated with non-persistence. We checked the proportional hazards assumption using Schoenfeld residuals, and this assumption was met (Newman, 2001).

All tests were carried out at the significance level of *α* = 0.05. The statistical software IBM SPSS for Windows, version 27, was used (IBM SPSS Inc., Armonk, NY, United States).

### Sensitivity Analyses

To evaluate the potential confounding caused by the inclusion of both new and prevalent antiplatelet medication users, we performed a stratified Cox regression analysis of factors associated with non-persistence separately in the two mentioned groups. To analyze the possible influence of the length of the treatment gap period used to identify non-persistence on the results of our study, a sensitivity analysis using shorter (1–5 months) and longer (12 months) treatment gaps was performed. Since the 5 years follow-up is a relatively long period of time, we identified factors associated with non-persistence in a sensitivity analysis with a shorter 3 years follow-up period.

## Results

The baseline characteristics of the study cohort (*n* = 9,178) are summarized in Table 1. During the first, second, third, fourth, and fifth year of the follow-up, 14.7, 7.3, 5.4, 3.8, and 1.8% of patients, respectively, became non-persistent with antiplatelet medications. At the end of the 5 years follow-up, 3,032 (33.0%) patients were identified as non-persistent with antiplatelet medications.

**TABLE 1:** Baseline characteristics of the study cohort.

Factor	All (*n* = 9,178)	Persistent (*n* = 6,146)	Non-persistent (*n* = 3,032)	*p*
Socio-demographic characteristics				
Age	75.2 ± 6.8	76.0 ± 7.1	73.7 ± 6.0	**<0.001** *
Female sex	5,285 (57.6)	3,413 (55.5)	1,872 (61.7)	**<0.001**
University education	637 (6.9)	393 (6.4)	244 (8.0)	**0.003**
Employed patients	448 (4.9)	258 (4.2)	190 (6.3)	**<0.001**
History of cardiovascular events^a^				
History of ischemic stroke	1,756 (19.1)	1,314 (21.4)	442 (14.6)	**<0.001**
History of TIA	715 (7.8)	488 (7.9)	227 (7.5)	0.446
History of MI	577 (6.3)	445 (7.2)	132 (4.4)	**<0.001**
Comorbid conditions				
Number of comorbid conditions	2.8 ± 1.6	2.9 ± 1.6	2.6 ± 1.6	**<0.001** *
Arterial hypertension	7,551 (82.3)	5,218 (84.9)	2,333 (76.9)	**<0.001**
Chronic heart failure	739 (8.1)	563 (9.2)	176 (5.8)	**<0.001**
Atrial fibrillation	1,124 (12.2)	736 (12.0)	388 (12.8)	0.259
Diabetes mellitus	3,866 (42.1)	2,739 (44.6)	1,127 (37.2)	**<0.001**
Hypercholesterolemia	3,577 (39.0)	2,361 (38.4)	1,216 (40.1)	0.118
Dementia	815 (8.9)	644 (10.5)	171 (5.6)	**<0.001**
Depression	1,082 (11.8)	745 (12.1)	337 (11.1)	0.159
Anxiety disorders	2,816 (30.7)	1,876 (30.5)	940 (31.0)	0.640
Parkinson’s Disease	444 (4.8)	326 (5.3)	118 (3.9)	**0.003**
Epilepsy	246 (2.7)	179 (2.9)	67 (2.2)	**0.049**
Bronchial asthma/COPD	2,106 (22.9)	1,397 (22.7)	709 (23.4)	0.484
Antiplatelet agent related characteristics				
Initial antiplatelet agent				
Aspirin	6,391 (69.6)	4,103 (66.8)	2,288 (75.5)	**<0.001**
Clopidogrel	1,562 (17.0)	1,121 (18.2)	441 (14.5)	
Ticlopidine	639 (7.0)	462 (7.5)	177 (5.8)	
Aspirin + clopidogrel	586 (6.4)	460 (7.5)	126 (4.2)	
New antiplatelet agent user^b^	1,314 (14.3)	737 (12.0)	577 (19.0)	**<0.001**
Patient´s co-payment (EUR)^c^	1.4 ± 1.2	1.5 ± 1.3	1.3 ± 1.1	<0.001*
General practitioner as index prescriber	6,678 (72.8)	4,626 (75.3)	2,052 (67.7)	**<0.001**
Cardiovascular co-medication				
Number of medications	8.1 ± 2.6	8.3 ± 2.5	7.8 ± 2.8	<0.001*
Number of CV medications	5.0 ± 2.3	5.1 ± 2.3	4.8 ± 2.3	<0.001*
Anticoagulants	1,917 (20.9)	1,300 (21.2)	617 (20.3)	0.374
Cardiac glycosides	744 (8.1)	578 (9.4)	166 (5.5)	**<0.001**
Antiarrhythmic agents	654 (7.1)	416 (6.8)	238 (7.8)	0.058
Beta-blockers	1,789 (19.5)	1,241 (20.2)	548 (18.1)	**0.016**
Thiazide diuretics	1,991 (21.7)	1,313 (21.4)	678 (22.4)	0.275
Loop diuretics	2,177 (23.7)	1,655 (26.9)	522 (17.2)	**<0.001**
Mineralocorticoid receptor antagonists	722 (7.9)	575 (9.4)	147 (4.8)	**<0.001**
Calcium channel blockers	2,856 (31.1)	1,918 (31.2)	938 (30.9)	0.792
RAAS inhibitors	7,659 (83.4)	5,188 (84.4)	2,471 (81.5)	**<0.001**
Statin	6,319 (68.8)	4,168 (67.8)	2,151 (70.9)	**0.002**
Lipid lowering agents other than statins^d^	902 (9.8)	596 (9.7)	306 (10.1)	0.550

In case of categorical variables, values represent the frequency and the percentages are provided in parentheses (% of *n*). In case of continuous variables, means ± standard deviations are provided. TIA–transient ischemic attack; MI–myocardial infarction; COPD–chronic obstructive pulmonary disease; CV–cardiovascular; RAAS–renin-angiotensin-aldosterone system; *p*–statistical significance between persistent and non-persistent patients according to the χ^2^-test; *Statistical significance according to the Mann-Whitney U test; In case of statistical significance (*p* < 0.05), the values are expressed in bold.

a The time period covered by “history”–5 years before the index date of this study.

b New antiplatelet agent user–patient in whom antiplatelet treatment was initiated in association with the diagnosis of peripheral arterial disease.

c Co-payment–calculated as the cost of antiplatelet treatment paid by the patient per month.

d Lipid lowering agents other than statins–ezetimibe and fibrates.

**FIGURE 2:**
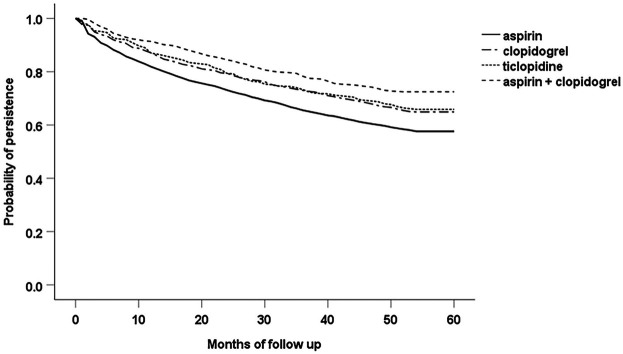
Persistence among the groups of patients with particular antiplatelet agents used at the index date of the study.

The Kaplan-Meier analysis revealed a significant difference (*p* < 0.001 according to the log-rank test) in persistence among the groups of patients created according to the particular antiplatelet agents used initially at the time of PAD diagnosis (aspirin, clopidogrel, ticlopidine and combination of aspirin with clopidogrel). The sharpest decline of the survival curve can be seen with aspirin, and the curve of patients with combination of aspirin and clopidogrel indicates the lowest likelihood of treatment discontinuation **(** Figure 2 **)**.

Table 2 shows the results of the Cox proportional hazards model which analyzed potential association between patient- and medication-related characteristics and non-persistence. Age, history of ischemic stroke or MI, clopidogrel or combination of aspirin and clopidogrel as the initial antiplatelet medication, higher patient’s co-payment, general practitioner as index prescriber and higher overall number of medications appeared as protective factors decreasing the patient’s likelihood of non-persistence. On the other hand, female sex, atrial fibrillation, anxiety disorders, bronchial asthma/chronic obstructive pulmonary disease (COPD), being a new antiplatelet medication user, use of anticoagulants or antiarrhythmic agents represented factors associated with increased probability of non-persistence.

**TABLE 2:** Multivariate analysis of the association between patient- and medication-related characteristics and the likelihood of non-persistence (*n* = 9,178).

Factor	Hr (95% CI)
Socio-demographic characteristics	
Age	**0.98 (0.97–0.98)**
Female sex	**1.26 (1.16–1.37)**
University education	1.10 (0.96–1.26)
Employed patients	1.13 (0.97–1.32)
History of cardiovascular events*^a^*	
History of ischemic stroke	**0.87 (0.78–0.97)**
History of TIA	1.08 (0.93–1.24)
History of MI	**0.82 (0.68–0.98)**
Comorbid conditions	
Number of comorbid conditions	0.92 (0.83–1.02)
Arterial hypertension	0.96 (0.83–1.11)
Chronic heart failure	1.03 (0.84–1.25)
Atrial fibrillation	**1.56 (1.33–1.84)**
Diabetes mellitus	0.89 (0.78–1.01)
Hypercholesterolemia	1.13 (0.99–1.29)
Dementia	0.84 (0.69–1.01)
Depression	1.07 (0.91–1.25)
Anxiety disorders	**1.17 (1.03–1.34)**
Parkinson’s Disease	1.02 (0.82–1.27)
Epilepsy	1.04 (0.79–1.36)
Bronchial asthma/COPD	**1.20 (1.04–1.37)**
Antiplatelet agent related characteristics	
Initial antiplatelet agent	
Aspirin	1.00
Clopidogrel	**0.83 (0.73–0.94)**
Ticlopidine	0.88 (0.73–1.07)
Aspirin + clopidogrel	**0.60 (0.49–0.74)**
New antiplatelet agent user^b^	**1.44 (1.28–1.62)**
Patient´s co-payment (EUR)^c^	**0.94 (0.89–0.98)**
General practitioner as index prescriber	**0.81 (0.74–0.88)**
Cardiovascular co-medication	
Number of medications	**0.95 (0.93–0.97)**
Number of CV medications	0.99 (0.95–1.03)
Anticoagulants	**1.15 (1.04–1.28)**
Cardiac glycosides	1.03 (0.86–1.22)
Antiarrhythmic agents	**1.33 (1.13–1.55)**
Beta-blockers	0.95 (0.86–1.06)
Thiazide diuretics	1.09 (0.99–1.20)
Loop diuretics	0.94 (0.83–1.05)
Mineralocorticoid receptor antagonists	0.84 (0.69–1.01)
Calcium channel blockers	1.07 (0.97–1.17)
RAAS inhibitors	1.02 (0.91–1.15)
Statin	1.01 (0.93–1.11)
Lipid lowering agents other than statins^d^	1.04 (0.92–1.18)

Values represent hazard ratios (95% confidence intervals). In case of statistical significance (*p* < 0.05), the values are expressed in bold. TIA–transient ischemic attack; MI–myocardial infarction; COPD–chronic obstructive pulmonary disease; CV–cardiovascular; RAAS–renin-angiotensin-aldosterone system.

a The time period covered by “history”–5 years before the index date of this study.

b New antiplatelet agent user–patient in whom antiplatelet treatment was initiated in association with the diagnosis of peripheral arterial disease.

c Co-payment–calculated as the cost of antiplatelet treatment paid by the patient per month.

d Lipid lowering agents other than statins–ezetimibe and fibrates.

### Sensitivity Analyses

The distribution of patient- and medication-associated characteristics in the groups of new and prevalent antiplatelet medication users is shown in Supplementary Table S3. Factors associated with non-persistence evaluated separately in the groups of new and prevalent antiplatelet medication users are listed in Supplementary Table S4. In the analysis performed in the group of prevalent users, almost the same factors associated with non-persistence as those in the main analysis, which included both new and prevalent users, were found. The only exceptions were: history of MI and anxiety disorders represented factors influencing persistence in the main cohort but not in the group of prevalent users, while mineralocorticoid receptor antagonists were associated with persistence only in the group of prevalent users but not in the main cohort. On the other hand, in the group of new users only age and the use of combination of aspirin and clopidogrel at the time of inclusion in the study were associated with persistence.

The inverse relationship between the length of the treatment gap period (1–6, 12 months) and the proportion of non-persistent patients was confirmed **(** Table 3 **)**. The 6 month period defining non-persistence may be considered as optimal choice since the use of shorter (1–5 months) or longer (12 months) treatment gap periods may lead to over- or underestimation of non-persistence. In the sensitivity analysis which used the Cox model with a shorter 3 years follow-up period, the same characteristics, except for hypercholesterolemia, as those in the main model with a 5 years follow-up were associated with non-persistence. Hypercholesterolemia was associated with non-persistence only in the Cox model with a 3 years follow up period but not in one with a 5 years follow-up **(** Supplementary Table S5 **)**.

**TABLE 3:** Sensitivity analysis of the effect of different lengths of treatment gap period defining non-persistence.

	Treatment gap to define non-persistence (months)						
	1	2	3	4	5	6	12
	Non-persistent patients						
1st year	3,897 (42.5)	2,861 (31.2)	2,187 (23.8)	1,804 (19.7)	1,524 (16.6)	1,350 (14.7)	832 (9.1)
2nd year	844 (9.2)	782 (8.5)	780 (8.5)	770 (8.4)	729 (7.9)	670 (7.3)	552 (6.0)
3rd year	333 (3.6)	445 (4.9)	476 (5.2)	498 (5.4)	493 (5.4)	498 (5.4)	441 (4.8)
4th year	190 (2.1)	296 (3.2)	340 (3.7)	333 (3.6)	346 (3.8)	353 (3.8)	344 (3.7)
5th year	140 (1.5)	221 (2.4)	223 (2.4)	202 (2.2)	194 (2.1)	161 (1.8)	18 (0.2)
Total	5,404 (58.9)	4,605 (50.2)	4,006 (43.6)	3,607 (39.3)	3,286 (35.8)	3,032 (33.0)	2,187 (23.8)

Values represent the frequency, and the percentages are provided in parentheses (% of *n* = 9,178).

## Discussion

Our study focused on the analysis of non-persistence with antiplatelet treatment in older PAD patients revealed some important findings. The proportion of PAD patients who became non-persistent with antiplatelet medication during the 5 years follow-up period (33.0%) can be considered as high. In the Cox regression model, factors associated with increased or decreased probability of non-persistence were identified. To the best of our knowledge, there are almost no similar studies evaluating the issue of non-persistence with antiplatelet medications used in secondary prevention of PAD in older patients. As we have mentioned previously in the Introduction, the only study similar to ours was the study by Qvist et al. (2019) which focused on adherence to antiplatelet and statin treatment in patients with PAD and abdominal aortic aneurysm. However, in contrast to our study, characteristics associated with non-persistence with antiplatelet medications were not found in that study. For the reasons mentioned above, we compared our results mostly with the studies analyzing persistence with antiplatelet treatment in patients after MI or stroke/TIA. The recommendations for the use of antiplatelet agents in recent guidelines of the European Society of Cardiology on the diagnosis and treatment of PAD by Aboyans et al. (2018) do not basically differ from those in the previous guideline by Tendera et al. (2011) which represented the actual guideline for the treatment of PAD at the time of inclusion of patients in our study.

Among socio-demographic characteristics evaluated in our study, higher age was associated with persistence, while female sex was associated with non-persistence. Better persistence in older PAD patients may indicate a careful medication-taking behavior in this age group of patients who are used to take concurrently several medications. Similarly to our study, in the systematic review by Jang and Zuniga (2020), older age was associated with persistence, while female sex was associated with non-persistence. That systematic review was focused on observational studies evaluating adult ischemic stroke or TIA patients, and reported poor persistence with antiplatelet agents, anticoagulants, and statins. Female sex was associated with poorer persistence also in the retrospective observational database study by Liu et al. (2019) which analyzed initiation of and persistence with antiplatelet medications among patients with acute coronary syndromes. Patients with no gaps of ≥ 30 days in antiplatelet treatment were considered persistent. The findings of our study do not make it possible to explain why female gender was associated with non-persistence. One possible factor contributing to the increased risk of non-persistence in women may be the fact that, in general, women experience adverse drug reactions (ADRs) more frequently than men (Miller, 2001; Franconi et al., 2007). ADRs may consequently lead to treatment discontinuation.

Certain comorbid conditions, namely atrial fibrillation, anxiety disorders and asthma/COPD as well as some CV co-medications like anticoagulants and antiarrhythmic agents were associated with non-persistence in our study cohort. Increased risk of bleeding associated with concurrent use of antiplatelet agents and anticoagulants may serve as one possible explanation of an increased likelihood of antiplatelet treatment discontinuation in older PAD patients with atrial fibrillation (Hindricks et al., 2021). According to a nationwide population-based cohort study by Green et al. (2016), atrial fibrillation was associated with a higher risk of treatment breaks in DAPT among patients undergoing percutaneous coronary intervention after an acute MI. Anxiety is associated with a fear of developing ADRs which may be responsible for treatment discontinuation (Sundbom and Bingefors, 2013). Anxiety has previously been reported to be a patient factor affecting adherence to medications in older adults in the systematic review by Yap et al. (2016). Patients who interrupted DAPT had a higher rate of some comorbidities including COPD in the study by Ferreira-González et al. (2012). Their study was focused on the analysis of risk of major cardiac events associated with discontinuation of DAPT in patients after drug-eluting stent implantation.

In our study cohort, PAD patients who initiated antiplatelet treatment with clopidogrel or DAPT (combination of aspirin and clopidogrel) had better persistence in comparison with those treated with aspirin alone. The positive association of DAPT with persistence may be related to its use after stenting in patients undergoing percutaneous coronary intervention after acute MI (Ibanez et al., 2018). History of MI and ischemic stroke represented factors associated with persistence in our study. This result may indicate better awareness of the importance of antiplatelet treatment in PAD patients with other conditions where antiplatelet medication is fully indicated in secondary prevention (Arnan et al., 2014; Kernan et al., 2014; Ibanez et al., 2018).

In our study, being a new user of antiplatelet medication appeared as a factor associated with non-persistence. Analogously, in the study by Qvist et al. (2019), among antiplatelet medication non-users at baseline, 57% persisted, whereas among users at baseline, 79% persisted with their antiplatelet therapy during the 5 years follow-up. After the first acute coronary syndrome-related hospitalization, patients who had prior use of antiplatelet agents were more likely to persist with this medication also in the study by Liu et al. (2019).

Higher co-payment was associated with persistence in our study cohort. The design of our study does not make it possible to explain this result. In contrast to our results, according to the claims database study by Burke et al. (2010), higher medication co-payment had a negative impact on persistence with antiplatelet therapy among ischemic stroke survivors. In their study, non-persistence was defined as an absence of medication refilling within 30 days from the run-out date of the prior prescription.

Increasing overall number of medications appeared as a protective factor associated with persistence in our study. Polypharmacy was associated with persistence also in our previous study focused on the analysis of non-persistence with antiplatelet medications in older patients after a TIA (Wawruch et al., 2017). In our study, index prescriber being a general practitioner rather than a specialist was associated with persistence. Similar association was found in our previous study focused on non-persistence with statins in older PAD patients (Wawruch et al., 2019).

Our study has some limitations which should be taken into consideration when interpreting its results. The database of the General Health Insurance Company, which served as a source of data for our study, was primarily constituted for insurance and reimbursement purposes and not for research. For this reason, it is impossible to identify the reasons for discontinuation and distinguish who decided to discontinue the treatment (i.e., the patient or the physician). Moreover, the database does not make it possible to identify whether patients actually took their medications as prescribed. We did not have access to data beyond the end of the study period. For this reason, it was impossible to identify the 6 months treatment gap during the period of less than 6 months before the end of the follow up. Inclusion of prevalent users in our study cohort (i.e., patients taking a therapy for a certain period of time before the index date of the study) can cause two types of biases: 1) prevalent users “survived” the early period of pharmacotherapy; this may cause selection bias, and 2) covariates of prevalent users at the time of inclusion in the study may be significantly affected by the drug itself (ENCePP Guide on Methodological Standards in Pharmacoepidemiology, 2020). Since PAD represents one of manifestations of atherosclerosis, which is a systemic process affecting the whole vasculature, PAD patients often use antiplatelet agents at the time of PAD diagnosis, e.g., because of coronary artery disease or after stroke/TIA. In our study, new users represented only 14.3% of the cohort of 9,178 patients. Exclusion of remaining 85.7% of patients would lead to a substantial confounding. This was the reason why we decided to prefer also to include prevalent users. On the other hand, the large sample size which covers all administrative regions of the Slovak Republic and detailed and precise data on drug dispensations and patients’ comorbid conditions represent the strengths of our study.

Despite the limitations mentioned above, our study revealed that younger patients, females, subjects in whom aspirin as monotherapy was administered at the index date, patients without history of MI or ischemic stroke, those with atrial fibrillation, anxiety disorders, bronchial asthma/COPD, subjects with lower overall number of medications and with lower co-payment, new antiplatelet medication users in whom this therapy was initiated in association with PAD diagnosis, and patients treated with anticoagulants or antiarrhythmic agents represent the groups of PAD patients in whom an increased likelihood of antiplatelet treatment discontinuation may be expected.

## Conclusion

Our study revealed a relatively high proportion of older PAD patients who discontinued antiplatelet treatment (33.0%) at some point during the 5 years follow-up. This finding indicates that non-persistence with antiplatelet medications in older PAD patients represents an important public health issue. Factors characterizing patients with an increased probability of non-persistence make it possible to identify patient groups that require increased attention aimed at the improvement of persistence with antiplatelet medication.

## Data Availability

The datasets presented in this article are not readily available because the data that support the findings of this study are available from the General Health Insurance Company but restrictions apply to the availability of these data, which were used under license for the current study, and so are not publicly available. Data are however available from the authors upon reasonable request and with permission of the General Health Insurance Company. Requests to access the datasets should be directed to MW, martin.wawruch@gmail.com.

## References

[B1] Aboyans V. Ricco J. B. Bartelink M. E. L. Björck M. Brodmann M. Cohnert T. (2018). ESC Scientific Document GroupESC Guidelines on the Diagnosis and Treatment of Peripheral Arterial Diseases, in Collaboration with the European Society for Vascular Surgery (ESVS): Document Covering Atherosclerotic Disease of Extracranial Carotid and Vertebral, Mesenteric, Renal, Upper and Lower Extremity arteriesEndorsed by: the European Stroke Organization (ESO)The Task Force for the Diagnosis and Treatment of Peripheral Arterial Diseases of the European Society of Cardiology (ESC) and of the European Society for Vascular Surgery (ESVS). Eur. Heart J. 39 (9), 763–816. 10.1093/eurheartj/ehx095 28886620

[B2] Agrawal K. Eberhardt R. T. (2015). Contemporary Medical Management of Peripheral Arterial Disease. Cardiol. Clin. 33 (1), 111–137. 10.1016/j.ccl.2014.09.010 25439335

[B3] Arnan M. K. Burke G. L. Bushnell C. (2014). Secondary Prevention of Stroke in the Elderly: Focus on Drug Therapy. Drugs Aging 31 (10), 721–730. 10.1007/s40266-014-0212-2 25212952

[B4] Bevan G. H. White Solaru K. T. (2020). Evidence-Based Medical Management of Peripheral Artery Disease. Atvb 40 (3), 541–553. 10.1161/ATVBAHA.119.312142 31996023

[B5] Bonaca M. P. Creager M. A. (2015). Pharmacological Treatment and Current Management of Peripheral Artery Disease. Circ. Res. 116 (9), 1579–1598. 10.1161/CIRCRESAHA.114.303505 25908730

[B6] Burke J. P. Sander S. Shah H. Zarotsky V. Henk H. (2010). Impact of Persistence with Antiplatelet Therapy on Recurrent Ischemic Stroke and Predictors of Nonpersistence Among Ischemic Stroke Survivors. Curr. Med. Res. Opin. 26 (5), 1023–1030. 10.1185/03007991003670563 20199138

[B7] Burnier M. (2019). Is There a Threshold for Medication Adherence? Lessons Learnt from Electronic Monitoring of Drug Adherence. Front. Pharmacol. 9, 1540. 10.3389/fphar.2018.01540 30687099 PMC6334307

[B8] De Geest S. Zullig L. L. Dunbar-Jacob J. Helmy R. Hughes D. A. Wilson I. B. (2018). ESPACOMP Medication Adherence Reporting Guideline (EMERGE). Ann. Intern. Med. 169 (1), 30–35. 10.7326/M18-0543 29946690 PMC7643841

[B9] ENCePP Guide on Methodological Standards in Pharmacoepidemiology. 8th revision (2020). http://www.encepp.eu/standards_and_guidances/methodologicalGuide4_2_3_1.shtml (Accessed April 15, 2021).

[B10] Ferreira-González I. Marsal J. R. Ribera A. Permanyer-Miralda G. García-Del Blanco B. Martí G. (2012). Double Antiplatelet Therapy after Drug-Eluting Stent Implantation. J. Am. Coll. Cardiol. 60 (15), 1333–1339. 10.1016/j.jacc.2012.04.057 22999716

[B11] Fowkes F. G. R. Rudan D. Rudan I. Aboyans V. Denenberg J. O. McDermott M. M. (2013). Comparison of Global Estimates of Prevalence and Risk Factors for Peripheral Artery Disease in 2000 and 2010: a Systematic Review and Analysis. The Lancet 382 (9901), 1329–1340. 10.1016/S0140-6736(13)61249-0 23915883

[B12] Franconi F. Brunelleschi S. Steardo L. Cuomo V. (2007). Gender Differences in Drug Responses. Pharmacol. Res. 55 (2), 81–95. 10.1016/j.phrs.2006.11.001 17129734

[B13] Green A. Pottegård A. Broe A. Diness T. G. Emneus M. Hasvold P. (2016). Initiation and Persistence with Dual Antiplatelet Therapy after Acute Myocardial Infarction: a Danish Nationwide Population-Based Cohort Study. BMJ Open 6 (5), e010880. 10.1136/bmjopen-2015-010880 PMC4874119 27173812

[B14] Guidelines for ATC Classification and DDD Assignment 2018. Oslo: WHO Collaborating Centre for Drug Statistics Methodology (2018). 283.

[B15] Hindricks G. Potpara T. Dagres N. Arbelo E. Bax J. J. Blomström-Lundqvist C. (2021). ESC Scientific Document Group.2020 ESC Guidelines for the Diagnosis and Management of Atrial Fibrillation Developed in Collaboration with the European Association for Cardio-Thoracic Surgery (EACTS). Eur. Heart J. 42 (5), 373–498. 10.1093/eurheartj/ehaa612 32860505

[B16] Ibanez B. James S. Agewall S. Antunes M. J. Bucciarelli-Ducci C. Bueno H. (2018). ESC Scientific Document Group.ESC Guidelines for the Management of Acute Myocardial Infarction in Patients Presenting with ST-Segment Elevation: The Task Force for the Management of Acute Myocardial Infarction in Patients Presenting with ST-Segment Elevation of the European Society of Cardiology (ESC). Eur. Heart J. 39 (2), 119–177. 10.1093/eurheartj/ehs215 28886621

[B17] ICD - 10th International Statistical Classification of Diseases and Related Health Problems, 10th Revision. Geneva: WHO (1992). 191.

[B18] Jang D. E. Zuñiga J. A. (2020). Factors Associated with Medication Persistence Among Ischemic Stroke Patients: a Systematic Review. Neurol. Res. 42 (7), 537–546. 10.1080/01616412.2020.1754640 32321382

[B19] Kernan W. N. Ovbiagele B. Black H. R. Bravata D. M. Chimowitz M. I. Ezekowitz M. D. (2014). Guidelines for the Prevention of Stroke in Patients with Stroke and Transient Ischemic AttackGuidelines for the Prevention of Stroke in Patients with Stroke and Transient Ischemic Attack: a Guideline for Healthcare Professionals from the American Heart Association/American Stroke Association. Stroke 45 (7), 2160–2236. 10.1161/str.0000000000000024 24788967

[B20] Liu X. He X. Wu J. Luo D. (2019). Initiation and Persistence with Antiplatelet Agents Among the Patients with Acute Coronary Syndromes: a Retrospective, Observational Database Study in China. Ppa Vol. 13, 2159–2169. 10.2147/PPA.S228065 PMC6925556 31908423

[B21] Miller M. A. (2001). Gender-Based Differences in the Toxicity of Pharmaceuticals-The Food and Drug Administration's Perspective. Int. J. Toxicol. 20 (3), 149–152. 10.1080/109158101317097728 11488556

[B22] Morley R. L. Sharma A. Horsch A. D. Hinchliffe R. J. (2018). Peripheral Artery Disease. BMJ 360, j5842. 10.1136/bmj.j5842 29419394

[B23] Newman S. C. (2001). Biostatistical Methods in Epidemiology. Chichester: Wiley, 382.

[B24] Qvist I. Søgaard R. Lindholt J. S. Lorentzen V. Hallas J. Frost L. (2019). Adherence to Prescribed Drugs Among 65-74 Year Old Men Diagnosed with Abdominal Aortic Aneurysm or Peripheral Arterial Disease in a Screening Trial: A VIVA Substudy. Eur. J. Vasc. Endovascular Surg. 57 (3), 442–450. 10.1016/j.ejvs.2018.09.023 30393062

[B25] Sundbom L. Bingefors K. (2013). The Influence of Symptoms of Anxiety and Depression on Medication Nonadherence and its Causes: a Population Based Survey of Prescription Drug Users in Sweden. Ppa 7, 805–811. 10.2147/PPA.S50055 23983459 PMC3751505

[B26] Tendera M. Aboyans V. Bartelink M. L. Baumgartner I. Clément D. Collet J. P. (2011). ESC Committee for Practice GuidelinesESC Guidelines on the Diagnosis and Treatment of Peripheral Artery Diseases: Document Covering Atherosclerotic Disease of Extracranial Carotid and Vertebral, Mesenteric, Renal, Upper and Lower Extremity Arteries: the Task Force on the Diagnosis and Treatment of Peripheral Artery Diseases of the European Society of Cardiology (ESC). Eur. Heart J. 32 (22), 2851–1906. 10.1093/eurheartj/ehr211 21873417

[B27] Vrijens B. De Geest S. Hughes D. A. Przemyslaw K. Demonceau J. Ruppar T. (2012). ABC Project Team.A New Taxonomy for Describing and Defining Adherence to Medications. Br. J. Clin. Pharmacol. 73 (5), 691–705. 10.1111/j.1365-2125.2012.04167.x 22486599 PMC3403197

[B29] Wawruch M. Wimmer G. Jr. Murin J. Paduchova M. Tesar T. Hlinkova L. (2019). Patient-Associated Characteristics Influencing the Risk for Non-persistence with Statins in Older Patients with Peripheral Arterial Disease. Drugs Aging 36 (9), 863–873. 10.1007/s40266-019-00689-2 31256366

[B28] Wawruch M. Zatko D. Wimmer G. Jr. Luha J. Wimmerova S. Kukumberg P. (2017). Non-persistence with Antiplatelet Therapy in Elderly Patients after a Transient Ischemic Attack. Aging Clin. Exp. Res. 29 (6), 1121–1127. 10.1007/s40520-017-0745-4 28284002

[B30] Xu H-Y. Yu Y-J. Zhang Q-H. Hu H-Y. Li M. (2020). Tailored Interventions to Improve Medication Adherence for Cardiovascular Diseases. Front. Pharmacol. 11, 510339. 10.3389/fphar.2020.510339 33364935 PMC7751638

[B31] Yap A. F. Thirumoorthy T. Kwan Y. H. (2016). Systematic Review of the Barriers Affecting Medication Adherence in Older Adults. Geriatr. Gerontol. Int. 16 (10), 1093–1101. 10.1111/ggi.12616 26482548

