# Supplementary material for: Non-Persistence With Antiplatelet Medications Among Older Patients With Peripheral Arterial Disease
Source: Front Pharmacol. 2021 May 19;12:687549. doi: 10.3389/fphar.2021.687549 (PMC8170080; doi:10.3389/fphar.2021.687549)
Supplement: Supplementary file 1 [file Table5.pdf]

Supplementary Table S5 Multivariate analysis of the association between patient- and medication-related characteristics and the likelihood of non-persistence with a 3-year follow-up period.

**Factor**	**HR (95% CI)**
Socio-demographic characteristics	
Age	**0.99 (0.98–0.99)**
Female sex	**1.22 (1.12–1.33)**
University education	1.04 (0.89–1.21)
Employed patients	1.12 (0.94–1.32)
History of cardiovascular events[a]	
History of ischemic stroke	**0.86 (0.77–0.97)**
History of TIA	1.04 (0.89–1.21)
History of MI	**0.78 (0.64–0.94)**
Comorbid conditions	
Number of comorbid conditions	0.91 (0.82–1.02)
Arterial hypertension	0.98 (0.84–1.15)
Chronic heart failure	0.99 (0.81–1.21)
Atrial fibrillation	**1.54 (1.30–1.82)**
Diabetes mellitus	0.95 (0.83–1.09)
Hypercholesterolemia	**1.18 (1.03–1.35)**
Dementia	0.92 (0.76–1.11)
Depression	1.09 (0.93–1.29)
Anxiety disorders	**1.19 (1.03–1.36)**
Parkinson's disease	1.12 (0.90 –1.40)
Epilepsy	1.12 (0.86–1.47)
Bronchial asthma/COPD	**1.20 (1.04–1.39)**

(Table continued)

Factor	HR (95% CI)
Antiplatelet agent related characteristics	
Initial antiplatelet agent	
Aspirin	1.00
Clopidogrel	**0.86 (0.75–0.98)**
Ticlopidine	1.00 (0.82–1.23)
Aspirin + Clopidogrel	**0.68 (0.55–0.83)**
New antiplatelet agent user[b]	**1.55 (1.37–1.74)**
Patient´s co-payment (EUR) [c]	**0.91 (0.86–0.95)**
General practitioner as index prescriber	**0.76 (0.69–0.83)**
Cardiovascular co-medication	
Number of medications	**0.94 (0.92–0.96)**
Number of CV medications	0.99 (0.95–1.03)
Anticoagulants	**1.16 (1.04–1.29)**
Cardiac glycosides	1.15 (0.98–1.36)
Antiarrhythmic agents	**1.28 (1.08–1.50)**
Beta-blockers	0.92 (0.82–1.03)
Thiazide diuretics	1.04 (0.94–1.15)
Loop diuretics	1.05 (0.93–1.19)
Mineralocorticoid receptor antagonists	0.89 (0.74–1.07)
Calcium channel blockers	1.04 (0.94–1.15)
RAAS inhibitors	1.08 (0.96–1.22)
Statin	0.97 (0.89–1.07)
Lipid lowering agents other than statins[d]	1.04 (0.91–1.20)

Values represent hazard ratios (95% confidence intervals). In case of statistical significance ($p<0.05$), the values are expressed in bold. TIA – transient ischemic attack; MI – myocardial infarction; COPD – chronic obstructive pulmonary disease; CV – cardiovascular; RAAS – renin

angiotensin aldosterone system. [a]The time period covered by “history” – 5 years before the index date of this study. [b]New antiplatelet agent user – patient in whom antiplatelet treatment was initiated in association with the diagnosis of peripheral arterial disease (PAD). [c]Co-payment – calculated as the cost of antiplatelet treatment paid by the patient per month. [d]Lipid lowering agents other than statins – ezetimibe and fibrates.
