# Supplementary material for: Non-Persistence With Antiplatelet Medications Among Older Patients With Peripheral Arterial Disease
Source: Front Pharmacol. 2021 May 19;12:687549. doi: 10.3389/fphar.2021.687549 (PMC8170080; doi:10.3389/fphar.2021.687549)
Supplement: Supplementary file 2 [file Table4.pdf]

Supplementary Table S4 Multivariate analysis of the association between patient- and medication-related characteristics and the likelihood of non-persistence in the groups of prevalent and new users.

**Factor**	**Prevalent users (n=7864)**	**New users (n=1314)**
Socio-demographic characteristics		
Age	**0.98 (0.97–0.99)**	**0.96 (0.95–0.98)**
Female sex	**1.30 (1.19–1.42)**	1.09 (0.90–1.32)
University education	1.05 (0.90–1.23)	1.31 (0.94–1.81)
Employed patients	1.13 (0.95–1.34)	1.07 (0.73–1.56)
History of cardiovascular events[a]		
History of ischemic stroke	**0.86 (0.77–0.96)**	1.10 (0.74–1.64)
History of TIA	1.08 (0.93–1.25)	0.99 (0.57–1.73)
History of MI	0.84 (0.70–1.01)	0.64 (0.28–1.45)
Comorbid conditions		
Number of comorbid conditions	0.94 (0.84–1.05)	0.89 (0.70–1.15)
Arterial hypertension	0.92 (0.78–1.09)	1.07 (0.78–1.48)
Chronic heart failure	1.02 (0.83–1.25)	0,90 (0.48–1.67)
Atrial fibrillation	**1.58 (1.32–1.88)**	1.41 (0.86–2.29)
Diabetes mellitus	0.88 (0.77–1.02)	0.86 (0.62–1.18)
Hypercholesterolemia	1.06 (0.92–1.23)	1.39 (0.99–1.93)
Dementia	0.82 (0.67–1.01)	0.71 (0.37–1.38)
Depression	1.03 (0.87–1.22)	1.17 (0.77–1.77)
Anxiety disorders	1.14 (0.98–1.31)	1.34 (0.97–1.87)
Parkinson’s disease	1.05 (0.83–1.33)	0.72 (0.39–1.35)
Epilepsy	1.06 (0.79–1.41)	0.98 (0.47–2.04)
Bronchial asthma/COPD	**1.19 (1.02-1.38)**	1.18 (0.83–1.68)
Antiplatelet agent related characteristics		
Initial antiplatelet agent		
Aspirin	1.00	1.00
Clopidogrel	**0.81 (0.70–0.94)**	0.86 (0.67–1.09)
Ticlopidine	0.86 (0.70–1.06)	1.26 (0.69–2.32)
Aspirin + clopidogrel	**0.60 (0.47–0.77)**	**0.57 (0.39–0.84)**
Patient´s co-payment (EUR)[b]	**0.93 (0.89–0.98)**	0.96 (0.87–1.07)
General practitioner as index prescriber	**0.78 (0.71–0.85)**	0.92 (0.76–1.11)
Cardiovascular co-medication		
Number of medications	**0.94 (0.92–0.97)**	0.98 (0.94–1.03)
Number of CV medications	1.00 (0.96–1.04)	0.96 (0.85–1.08)
Anticoagulants	**1.15 (1.03–1.29)**	1.02 (0.74–1.41)
Cardiac glycosides	0.99 (0.82–1,19)	1.43 (0.78–2.61)
Antiarrhythmic agents	**1.33 (1.13–1.57)**	1.25 (0.70–2.23)
Beta-blockers	0.91 (0.81–1.02)	1.26 (0.95–1.67)

(Table continued)

Factor	Prevalent users (n=7864)	New users (n=1314)
Thiazide diuretics	1.09 (0.98–1.21)	1.02 (0.78–1.34)
Loop diuretics	0.94 (0.83–1.06)	0.86 (0.59–1.24)
Mineralocorticoid receptor antagonists	**0.79 (0.65–0.96)**	1.35 (0.74–2.47)
Calcium channel blockers	1.04 (0.94–1.16)	1.16 (0.91–1.47)
RAAS inhibitors	1.08 (0.95–1.22)	0.89 (0.68–1.15)
Statin	1.03 (0.93–1.14)	1.00 (0.81–1.24)
Lipid lowering agents other than statins[c]	1.03 (0.90–1.18)	1.07 (0.77–1.49)

Values represent hazard ratios (95% confidence intervals). In case of statistical significance ($p<0.05$), the values are expressed in bold. TIA – transient ischemic attack; MI – myocardial infarction; COPD – chronic obstructive pulmonary disease; CV – cardiovascular; RAAS – renin angiotensin aldosterone system. [a]The time period covered by “history” – 5 years before the index date of this study. [b]Co-payment – calculated as the cost of antiplatelet treatment paid by the patient per month. [c]Lipid lowering agents other than statins – ezetimibe and fibrates.
