# Supplementary material for: Non-Persistence With Antiplatelet Medications Among Older Patients With Peripheral Arterial Disease
Source: Front Pharmacol. 2021 May 19;12:687549. doi: 10.3389/fphar.2021.687549 (PMC8170080; doi:10.3389/fphar.2021.687549)
Supplement: Supplementary file 3 [file Table1.pdf]

Supplementary Table S1 Codes of diseases according to the ICD – 10th International Statistical Classification of Diseases and Related Health Problems (1992).

**Disease**	**ICD-10 codes**
Peripheral arterial disease	I70.21 – I70.24
Ischemic stroke	I63
Transient ischemic attack	G45
Myocardial infraction	I21, I22
Arterial hypertension	I10
Chronic heart failure	I50
Atrial fibrillation	I48.0 –I48.2
Diabetes mellitus	E10, E11
Hypercholesterolemia	E78.0, E78.2, E78.4
Dementia	F00, F01, F02, F03, G30
Depression	F32, F33
Anxiety disorders	F40, F41
Parkinson’s disease	G20
Epilepsy	G40
Bronchial asthma	J45
Chronic obstructive pulmonary disease	J44
