# Supplementary material for: Non-Persistence With Antiplatelet Medications Among Older Patients With Peripheral Arterial Disease
Source: Front Pharmacol. 2021 May 19;12:687549. doi: 10.3389/fphar.2021.687549 (PMC8170080; doi:10.3389/fphar.2021.687549)
Supplement: Supplementary file 4 [file Table2.pdf]

Supplementary Table S2 Codes of medications according to Guidelines for ATC classification and DDD assignment (2018).

Medication	ATC codes
Initial antiplatelet agent	
Aspirin	B01AC06
Clopidogrel	B01AC04
Ticlopidine	B01AC05
Anticoagulants	B01AA, B01AB, B01AE, B01AF
Cardiac glycosides	C01AA
Antiarrhythmic agents	C01BA–D
Beta-blockers	C07A, C07B*, C07FB*
Thiazide diuretics	C03AA, C03EA01, C07B*, C09BA*, C09DA*, C09DX01*, C09DX03*, C09XA52*
Loop diuretics	C03CA
Mineralocorticoid receptor antagonists	C03DA
Calcium channel blockers	C08CA, C08DA–B, C07FB*, C09DB*, C09DX03*, C10BX03*, C10BX09*, C10BX11*, C10BX14*
RAAS inhibitors	C09A, C09BA*, C09C, C09DA*, C09DB*, C09DX01*, C09DX03*, C09XA02, C09XA52*, C10BX10*, C10BX11*, C10BX14*, C10BX15*
Statin	C10AA, C10BA02*, CA10BA03*, C10BA04*, C10BA05*, C10BA06*, C10BX03*, C10BX09*, C10BX10*, C10BX11*, C10BX14*, C10BX15*
Fibrates	C10AB, CA10BA03*, C10BA04*
Ezetimibe	C10AX09, C10BA02*, C10BA05*, C10BA06*

*combination preparation
