# Supplementary material for: Non-Persistence With Antiplatelet Medications Among Older Patients With Peripheral Arterial Disease
Source: Front Pharmacol. 2021 May 19;12:687549. doi: 10.3389/fphar.2021.687549 (PMC8170080; doi:10.3389/fphar.2021.687549)
Supplement: Supplementary file 5 [file Table3.pdf]

Supplementary Table S3 The distribution of patient- and medication-associated characteristics in the groups of new and prevalent antiplatelet medication users

Factor	Prevalent users				New users			
	All (n=7864)	Persistent (n=5409)	Non-persistent (n=2455)	p	All (n=1314)	Persistent (n=737)	Non-persistent (n=577)	p
Socio-demographic characteristics								
Age	75.4 ± 6.8	76.1 ± 7.1	74.0 ± 6.1	**<0.001**	73.9 ± 6.6	75.1 ± 7.0	72.4 ± 5.6	**<0.001**
Female sex	4610 (58.6)	3053 (56.4)	1557 (63.4)	**<0.001**	675 (51.4)	360 (48.8)	315 (54.6)	**0.039**
University education	547 (7.0)	354 (6.5)	193 (7.9)	**0.033**	90 (6.8)	39 (5.3)	51 (8.8)	**0.012**
Employed patients	384 (4.9)	234 (4.3)	150 (6.1)	**0.001**	64 (4.9)	24 (3.3)	40 (6.9)	**0.002**
History of cardiovascular events[a]								
History of ischemic stroke	1664 (21.2)	1253 (23.2)	411 (16.7)	**<0.001**	92 (7.0)	61 (8.3)	31 (5.4)	**0.041**
History of TIA	679 (8.6)	467 (8.6)	212 (8.6)	0.998	36 (2.7)	21 (2.8)	15 (2.6)	0.783
History of MI	550 (7.0)	425 (7.9)	125 (5.1)	**<0.001**	27 (2.1)	20 (2.7)	7 (1.2)	0.057
Comorbid conditions								
Number of comorbid conditions	3.0 ± 1.6	3.0 ± 1.6	2.8 ± 1.6	**<0.001**	2.0 ± 1.6	2.0 ± 1.6	1.9 ± 1.5	0.287
Arterial hypertension	6871 (87.4)	4812 (89.0)	2059 (83.9)	**<0.001**	680 (51.8)	406 (55.1)	274 (47.5)	**0.006**
Chronic heart failure	694 (8.8)	533 (9.9)	161 (6.6)	**<0.001**	45 (3.4)	30 (4.1)	15 (2.6)	0.146
Atrial fibrillation	1036 (13.2)	683 (12.6)	353 (14.4)	**0.033**	88 (6.7)	53 (7.2)	35 (6.1)	0.418
Diabetes mellitus	3417 (43.5)	2468 (45.6)	949 (38.7)	**<0.001**	449 (34.2)	271 (36.8)	178 (30.8)	**0.025**
Hypercholesterolemia	3199 (40.7)	2172 (40.2)	1027 (41.8)	0.160	378 (28.8)	189 (25.6)	189 (32.8)	**0.005**
Dementia	761 (9.7)	602 (11.1)	159 (6.5)	**<0.001**	54 (4.1)	42 (5.7)	12 (2.1)	**0.001**
Depression	980 (12.5)	690 (12.8)	290 (11.8)	0.240	102 (7.8)	55 (7.5)	47 (8.1)	0.646
Anxiety disorders	2520 (32.0)	1719 (31.8)	801 (32.6)	0.456	296 (22.5)	157 (21.3)	139 (24.1)	0.230
Parkinson’s disease	392 (5.0)	288 (5.3)	104 (4.2)	**0.040**	52 (4.0)	38 (5.2)	14 (2.4)	**0.012**

(Table continued)

Factor	Prevalent users				New users			
	All (n=7864)	Persistent (n=5409)	Non-persistent (n=2455)	p	All (n=1314)	Persistent (n=737)	Non-persistent (n=577)	p
Epilepsy	217 (2.8)	159 (2.9)	58 (2.4)	0.148	29 (2.2	20 (2.7)	9 (1.6)	0.158
Bronchial asthma/COPD	1885 (24.0)	1282 (23.7)	603 (24.6)	0.407	221 (16.8)	115 (15.6)	106 (18.4)	0.183
Antiplatelet agent related characteristics								
Initial antiplatelet agent								
Aspirin	5592 (71.1)	3696 (68.3)	1896 (77.2)	**<0.001**	799 (60.8)	407 (55.2)	392 (67.9)	**<0.001**
Clopidogrel	1238 (15.7)	925 (17.1)	313 (12.7)		324 (24.7)	196 (26.6)	128 (22.2)	
Ticlopidine	612 (7.8)	450 (8.3)	162 (6.6)		27 (2.1)	12 (1.6)	15 (2.6)	
Aspirin + clopidogrel	422 (5.4)	338 (6.2)	84 (3.4)		164 (12.5)	122 (16.6)	42 (7.3)	
Patient´s co-payment (EUR)[b]	1.4 ± 1.2	1.5 ± 1.3	1.3 ± 1.1	**<0.001**	1.4 ± 1.1	1.4 ± 1.2	1.3 ± 1.0	**0.004**
General practitioner as index prescriber	6069 (77.2)	4251 (78.6)	1818 (74.1)	**<0.001**	609 (46.3)	375 (50.9)	234 (40.6)	**<0.001**
Cardiovascular co-medication								
Number of medications	8.5 ± 2.3	8.6 ± 2.2	8.3 ± 2.4	**<0.001**	5.4 ± 3.1	5.6 ± 3.1		**0.020**
Number of CV medications	5.2 ± 2.3	5.3 ± 2.3	5.0 ± 2.2	**<0.001**	3.8 ± 2.0	3.9 ± 2.1	3.7 ± 2.0	**0.032**
Anticoagulants	1713 (21.8)	1173 (21.7)	540 (22.0)	0.758	204 (15.5)	127 (17.2)	77 (13.3)	0.053
Cardiac glycosides	689 (8.8)	539 (10.0)	150 (6.1)	**<0.001**	55 (4.2)	39 (5.3)	16 (2.8)	**0.024**
Antiarrhythmic agents	616 (7.8)	395 (7.3)	221 (9.0)	**0.009**	38 (2.9)	21 (2.8)	17 (2.9)	0.917
Beta-blockers	1623 (20.6)	1153 (21.3)	470 (19.1)	**0.027**	166 (12.6)	88 (11.9)	78 (13.5)	0.393
Thiazide diuretics	1788 (22.7)	1200 (22.2)	588 (24.0)	0.083	203 (15.4)	113 (15.3)	90 (15.6)	0.895
Loop diuretics	2015 (25.6)	1539 (28.5)	476 (19.4)	**<0.001**	162 (12.3)	116 (15.7)	46 (8.0)	**<0.001**
Mineralocorticoid receptor antagonists	676 (8.6)	544 (10.1)	132 (5.4)	**<0.001**	46 (3.5)	31 (4.2)	15 (2.6)	0.116
Calcium channel blockers	2541 (32.3)	1751 (32.4)	790 (32.2)	0.866	315 (24.0)	167 (22.7)	148 (25.6)	0.208
RAAS inhibitors	6714 (85.4)	4641 (85.8)	2073 (84.4)	0.113	945 (71.9)	547 (74.2)	398 (69.0)	**0.036**

(Table continued)

Factor	Prevalent users				New users			
	**All (n=7864)**	**Persistent (n=5409)**	**Non-persistent (n=2455)**	**p**	**All (n=1314)**	**Persistent (n=737)**	**Non-persistent (n=577)**	**p**
Statin	5458 (69.4)	3690 (68.2)	1768 (72.0)	**0.001**	861 (65.5)	478 (64.9)	383 (66.4)	0.565
Lipid lowering agents other than statins [c]	797 (10.1)	539 (10.0)	258 (10.5)	0.459	105 (8.0)	57 (7.7)	48 (8.3)	0.698

In case of categorical variables, values represent the frequency and the percentages are provided in parentheses (% of n). In case of continuous variables, means ± standard deviations are provided. TIA – transient ischemic attack; MI – myocardial infarction; COPD – chronic obstructive pulmonary disease; CV – cardiovascular; RAAS – renin-angiotensin-aldosterone-system; p – statistical significance between persistent and non-persistent patients according to the χ^2-test; * statistical significance according to the Mann-Whitney U test; in case of statistical significance ($p<0.05$), the values are expressed in bold. [a]The time period covered by "history" – 5 years before the index date of this study. [b]Co-payment – calculated as the cost of antiplatelet treatment paid by the patient per month. [c]Lipid lowering agents other than statins – ezetimibe and fibrates.
